# Multivariate Multi-Objective Allocation in Stratified Random Sampling: A Game Theoretic Approach

**DOI:** 10.1371/journal.pone.0167705

**Published:** 2016-12-09

**Authors:** Yousaf Shad Muhammad, Ijaz Hussain, Alaa Mohamd Shoukry

**Affiliations:** 1 Department of Statistics, Quaid-i-Azam University, Islamabad, Pakistan; 2 Arriyadh Community College, King Saud University Riyadh Saudi Arabia; King Saud University, KSA workers University, Egypt; Southwest University, CHINA

## Abstract

We consider the problem of multivariate multi-objective allocation where no or limited information is available within the stratum variance. Results show that a game theoretic approach (based on weighted goal programming) can be applied to sample size allocation problems. We use simulation technique to determine payoff matrix and to solve a minimax game.

## Introduction

A choice of sampling plan is fundamental to any statistical study because it provides estimates of population parameters. Sample size allocation to each stratum is necessary in stratified random sampling design. An optimum allocation can be applied to each characteristic unless sufficient information about stratum variability is available. However, the optimization technique can lead to misleading results because of limited information about cost and variance. The precision (1/variance) and cost may achieve in the process of implementation. For more discussion on it see [1–3], [4, 5] and [6].

In this study, we propose a multivariate game theoretic approach for the sample size allocation problem in stratified random sampling design. There are many techniques which are being used for allocation of sample size, such as proportional allocation, optimal allocation etc. All these techniques are suitable when we have sampling frame and other relevant information regarding population variance etc. These techniques are helpful and might be more relevant if we have sufficient information about population. In case when we have i) no or limited information about population ii) one cannot be much optimistic about the sample results that they will be true on average iii) there may be a high variability among sampling units iv) one wants to deal with adverse case scenario regarding variation in sample This technique will help him to answer the above question.

In such games proportional allocation technique is computationally feasible and generally applied ([7]). Within stratum Variance is vital for a game theoretic approach. In univariate, [8] formulated a mini-max allocation problem which is a function of specified minimum upper bounds for each stratum variances.

The [9] presented “a game theoretic formulation for the multivariate case, where the covariances between pairs of responses are supposed to be constant from stratum to stratum”. Moreover, these strategies are functions of stratum variance and covariances. The [10] discussed an optimum allocation for a multivariate design that “minimizes the cost of obtaining estimates with smaller errors than previously specified numbers with confidence level”. He also showed that variance information could be useful to obtain nearly optimum allocation.

The [11] obtained posterior variances by using the priori information of both, mean and variance. [12] and [13] proposed a “method of allocation in multivariate surveys where various stratum variances are assumed to be known”. It minimizes the cost of having estimates of variances smaller than its predecessor.

In game theory literature, many authors discussed various models of two players game. A traveler’s dilemma game (TDG) model on two coupled latices presented by [14] which investigate effects of coupling on cooperation. A simulation study of this model indicates that cooperation behavior varies over lattices. A two player game between cooperator and defector was discussed in [15] in which they simulated utility coupling on weighted lattice. An other two player game on a square lattices using different weights for available strategies modeled in [16]. A risk aversion model presented in [17] when player’s participation is probabilistic. The [18] modeled a two player game which considers the reputation and behavior diversity which varies over strategy space. Simulation results show that cooperation behavior influenced by reputation index.

Allocation in multivariate surveys must be optimum for all characteristics. For example, any such allocation which minimizes the cost vectors or the variance functions, which minimizes it or maximizes the relative efficiency comparing with other allocation. A detailed discussion given in [19] [20–22], [23–26], [27–30], [31] and [32]

Second section explains the sampling notations. We set a multi-objective game allocation problem in section 3. Section 4 explains methodology of our approach and discussion on results is given in Section 5.

## Sampling notations

### Population

Let we have population of size *N* which is further divided into *L* mutually exclusive strata, where $N=\sum_{h=1}^{L}N_{h}$. Consider a data set *Y*_*jhi*_ for *j* = 1, 2, …, *Q* characteristics and *h* = 1, 2, …, *L* strata with *i* = 1, 2, 3, … *N*_*h*_ sampling units in the *h*^*th*^ stratum. ${\bar{Y}}_{jh}=\sum_{i=1}^{N_{h}}\frac{Y_{jhi}}{N_{h}}$ is the population mean of *h*^*th*^ stratum of *j*^*th*^ characteristic.

If $W_{h}\;=\;\frac{N_{h}}{N}$ is weight of *h*^*th*^ stratum and $S_{jh}^{2}$ is the population variance of *j*^*th*^ study variable which can calculated from *h*^*th*^ stratum as;

$$\begin{array}{c} S_{jh}^{2}=\frac{1}{N_{h}-1}\sum_{i=1}^{N_{h}}{\left(Y_{jhi}-{\bar{Y}}_{jh}\right)}^{2} \end{array}$$

### 0.1 Sample

We draw a simple random sample of size *n*_*h*_ independently from each stratum such that $\sum_{h=1}^{L}n_{h}=n$. Let ${\bar{y}}_{jst}$ is stratified estimator of population mean ${\bar{Y}}_{j}$ of characteristic *j*, which is given as:

$${\bar{y}}_{jst}=\sum_{h=1}^{L}W_{h}{\bar{y}}_{jh}$$

where ${\bar{y}}_{jh}=\sum_{i=1}^{n_{h}}\frac{y_{jhi}}{n_{h}}$. The variance of ${\bar{y}}_{jst}$ is:

$$V\left({\bar{y}}_{jst}\right)=\sum_{h=1}^{L}\left(\frac{1}{n_{h}}-\frac{1}{Nh}\right){W_{h}}^{2}S_{jh}^{2}$$

Ignoring the term independent of *n*_*h*_, we have;

$$V\left({\bar{y}}_{jst}\right)=\sum_{h=1}^{L}\frac{{W_{h}}^{2}S_{jh}^{2}}{n_{h}}$$ (1)

If our interest lies in squared coefficient of variation instead of variance, we can use the following expression;

$$CV_{j}^{2}=\frac{V({\bar{y}}_{st,j})}{{\bar{Y}}_{j}^{2}}$$

where, ${\bar{Y}}_{j}=\sum_{h=1}^{L}W_{h}{\bar{Y}}_{jh}$. Substituting the value of $V({\bar{y}}_{jst})$ from Eq 1 in the above equation, we have;

$$CV_{j}^{2}=\sum_{h=1}^{L}\frac{W_{h}^{2}S_{jh}^{2}}{n_{h}{\bar{Y}}_{j}^{2}}$$ (2)

## Game setting in a multi-objective allocation problem

We draw a simple random sample from all strata such that $\sum_{h=1}^{L}n_{h}=n$ while assuming a finite population. The objective is to minimize some vector relation of coefficients of variation (CV) while allocating a sample in all strata. For a single characteristic, say *j*, the simple mean estimator of CV can be expressed as Eq (2).

In particular, an optimum allocation of a sample of size *n* is a choice of the *n*_*h*_ that minimizes Eq (2) subject to the restriction that $\sum_{h=1}^{L}n_{h}=n$ if values $S_{h}^{2}$ and ${\bar{Y}}_{j}$ are known. An optimum allocation only be computed if $S_{jh}^{2}$ and ${\bar{Y}}_{j}$ are known ([33]). We can use unbiased estimators as;

$$s_{jh}^{2}=\frac{1}{n_{h}-1}\sum_{i=1}^{n_{h}}{\left(y_{jhi}-{\bar{y}}_{jh}\right)}^{2}$$

and ${\bar{y}}_{jst}$ of $S_{jh}^{2}$ and ${\bar{Y}}_{j}$, respectively. Let say *z*_*jh*_ is *CV*^2^ that can be computed from sample as;

$$z_{jh}=cv_{jh}^{2}=\frac{s_{jh}^{2}}{n_{h}{\bar{y}}_{jh}^{2}}.$$ (3)

### Players: Sampler (player 1) and Adversary (player 2)

If we consider sampler as player 1, the *z*_*jh*_ from Eq (3) to be his loss in a zero-sum game against Adversary (player 2) for characteristic *j* in the stratum *h*. The sampler seeks an allocation that is a good strategy for playing this game to minimize some vector ${\vec{Z}}_{h}$ for all (*h* = 1, 2, …, *L*). The vector space of strategies (allocations) which are available to the sampler is considered to be *ν* is;

$$\nu =\{\vec{n}=\left[n_{1},n_{2},n_{3},\ldots ,n_{L}\right]:\sum_{h=1}^{L}n_{h}=n\}.$$ (4)

Therefore, the Adversary selects an independent sample from each strata according to an offered strategy by the sampler. The objective of the Adversary is to choose vector $\vec{Z_{h}}=\left[z_{1h},z_{2h},z_{3h},...,z_{Qh}\right]$ from each strata *hε*(1, 2, 3, …, *L*), which maximize, say;

$$Maximize\left(z_{1h},z_{2h},z_{3h},\ldots ,z_{Qh}\right)=Z_{h}.$$

A seemingly natural way to proceed which may lead to interesting results. The Adversary’s strategies are multi-objective goal program subject to maximize vector of $\vec{z_{h}}$ with in each stratum for a particular *n*_*h*_, *hε*(1, 2, 3, …, *L*). The Adversary’s strategy space Δ can be described as;

$$\Delta =\{z_{h},h\varepsilon \left(1,2,3,\ldots ,L\right):\;Z=\sum_{h=1}^{L}z_{h}\}.$$ (5)

### Payoff matrix of Sampler (player 1)

While playing a zero sum game, each player try to optimize his gain or loss. The minimax idea is minimizing the possible loss for a worst case (maximum loss) scenario. A minimax strategy is a mixed strategy game. Both players choose alternate strategies and they make simultaneous moves. It can also been extended to more complex games.

Sampler would like to minimize vector $\vec{Z}=\left[z_{1},z_{2},z_{3},...,z_{h}\right]$, where *z*_*h*_ is defined above. Payoff of sampler is the gain of Adversary, which can be determined by following multi-objective program;

$$\left.\begin{array}{c} \;\text{Maximize}\;\;z_{h}=\left(z_{1h},z_{2h},z_{3h},\ldots ,z_{Qh}\right):\;\forall \;h=1,2,...,L.\;\\ \;\text{Subject}\;\text{to}\;\\ 2\leq n_{h}\leq N_{h}\;\\ n_{h}\;\text{are}\;\text{integers},\;\forall \;h=1,2,\ldots ,L.\;\text{and}\;\;\vec{n}\varepsilon \nu . \end{array}\right\}$$ (6)

This can be equivalently written in a matrix Σ_*ν*×Δ_. Each row in Σ represents loss of sampler for a possible allocation and each column of Σ represents gain of Adversary for an offered strategy from sampler.

### Minimax game for allocation

Assume that the sampler and the Adversary each choose a strategy. This implies that the sampler will pick an allocation vector $\vec{n}=\left[n_{1},n_{2},n_{3},...,n_{L}\right]\;\varepsilon \;\nu :\;\sum_{h=1}^{L}n_{h}=n$ and the Adversary has to pick a sample of actual data according to Eq (6).

Adversary will choose a strategy that maximize *z*_*h*_ = (*z*_1*h*_, *z*_2*h*_, *z*_3*h*_, …, *z*_*Qh*_): ∀ *h* = 1, 2, …, *L*. Therefore, the sampler objective is to minimizes his maximum (worst) value within the available budget, while allocating sample of size *n* to all strata. However, obviously a larger sample will produce better result if there is no restriction on budget. The optimal program consider all possible choices of sample, where adversary can choose his strategies independently. In summary, the objective of the sampler under budgetary condition is;

$$\left.\begin{array}{c} \;\;\text{Minimize}\underset{\vec{n}\;\varepsilon \;\nu }{\;}\;\left(\text{Maximize}\underset{j\;\varepsilon \;Q}{\;}z_{h}=\left(z_{1h},z_{2h},z_{3h},\ldots ,z_{Qh}\right):\forall \;h=1,2,\ldots ,L.\right)\\ \;\;\text{Subject}\;\text{to}\\ \;2\leq n_{h}\leq N_{h}\\ \;c_{0}+\sum_{h=1}^{L}c_{h}n_{h}\leq C\\ \;\sum_{h=1}^{L}n_{h}=n\\ \;n_{h}\;\text{are}\;\text{integers}\;\;\forall \;h=1,2,\ldots ,L\;\text{and}\;\vec{n}\;\varepsilon \;\nu . \end{array}\right\}$$ (7)

***Theorem***: In the game described above, i.e., (*ν*, Δ, *CV*^2^)

- A good strategy for Adversary is $z_{h}^{*}=\sum_{j=1}^{Q}\;max\left(cv_{jh}^{2}:n_{h}\right)$
- A good strategy for the sampler is $CV^{2}=\sum_{h=1}^{L}\frac{W_{h}^{2}S_{h}^{2}}{n_{h}{\bar{y}}^{2}}$.
- An optimal solution *Z* exists in the allocation problem game, as described in program Eq (7)
  $$Z=\;\text{Minimize}\underset{\vec{n}\;\varepsilon \;\nu }{\;}\;\left(\text{Maximize}\underset{j\;\varepsilon \;Q}{\;}z_{h}=\left(z_{1h},z_{2h},z_{3h},\ldots ,z_{Qh}\right):\;\forall \;h=1,2,\ldots ,L\right)$$
  where Z is the value of the game.

## Solution of the allocation game

The solution of the allocation problem can be formulated, as in previous section. The idea is to understand the structure of the problem that will enable us to extend it into more complex cases. Consider the sampler’s problem from Eq (7). For some $\vec{n}=\left[n_{1},n_{2},n_{3},\ldots ,n_{L}\right]\;\varepsilon \;\nu$, the inner maximization problem given in Eq (6) is solved using any suitable goal programming technique ([22], [23], [28–31], [19, 34] and [35]).

The Adversary computes the maximum weighted sum from all characteristics (*j* = 1, 2, …, *Q*) using Eq (3) and model (6). This exercise is repeated for all strata (*h* = 1, 2, …, *L*).

We allow our generic goal program to have *Q* goals, which may be *j* = 1, …, *Q*. We determine *n*_*h*_ decision variables. These are the factors over which the decision maker(s) may control and determine the decisions to be made. Each goal has an achieved value, *z*_*jh*_, on its underlying criterion. *z*_*jh*_ is a function of the compromise decision variables for *j*^*th*^ goal. The whole situation is expressed below:

$$\left.\begin{array}{c} \text{Maximize}\;z_{h}\;=\;\sum_{j=1}^{Q}\;f_{jh}\left(n_{h}\right)\;\\ \text{Subject}\;\text{to}\;\\ 2\leq n_{h}\leq N_{h}\;\\ n_{h}\;\text{are}\;\text{integers}\;\;\forall \;h=1,2,\ldots ,L\;\text{and}\;j=1,2,\ldots ,Q. \end{array}\right\}$$ (8)

The above program can be expressed as a Weighted Goal Programming (WGP) if *f*_1*h*_, *f*_2*h*_, ⋯, *f*_*Qh*_ represent weighted functions in their respective priority. The WGP is formulated to maximize a composite objective function as a vector formed by a weighted sum of coefficients of variation in the respective strata.

The optimal strategy for the sampler is $\vec{n}=\left[n_{1},n_{2},n_{3},\ldots ,n_{L}\right]\;\varepsilon \;\nu$ using model (7). This implies for any strategy that the sampler would choose, as the Adversary will sample from every strata to maximize the model (6). Therefore, it is a minimal sampling scheme.

## Numerical Illustration

This idea of sample selection is applied on a real data of Master of Philosophy (Table 1) induction into the department of Statistics, Quiad-e-Azam university Islamabad, Fall 2014. Stratum 1 compose on ‘other universities’ inductees and stratum 2 QAU graduates inductees. Data below represent the ‘test plus interview’ marks and ‘academic record’ marks. A stratified random sample is desired to be selected from the given data. The cost of selecting a sampling unit from stratum 1 is Rs. 2000 and from stratum 2 is Rs. 1000 (estimate of the traveling cost in local units, for sampling purpose only). Let we have a budget of Rs. 15000 only, and there is no initial cost on sampling i.e., *C*_0_ = 0.

**Table 1:** Test and Interview marks Master of Philosophy, Dept. of Statistics, QAU Islamabad, Fall 2014.

Stratum 1		Stratum 2	
T & I	Ac. Rec.	T & I	Ac. Rec.
44	36	42	30
50	29	38	29
46	31	38	28
46	26	40	26
44	27	36	28
44	26	38	26
40	29	34	29
42	27	34	29
36	29	30	30
34	26		
30	28		

### Computation of payoff matrix

We use the model (6) to compute payoff matrix. Let the two characteristics be the test and interview marks (T & I) and academic record marks (Ac. Rec.). Both have the equal importance because total marks considered are in the selection as the criteria. The above model (6) can be represented as;

$$\left.\begin{array}{c} \;\text{Maximize}\;\;z_{h}=\frac{s_{1h}^{2}}{n_{h}{\bar{y}}_{1h}^{2}}+\frac{s_{2h}^{2}}{n_{h}{\bar{y}}_{2h}^{2}},h=1\;\text{and}\;2\;\\ \;\text{Subject}\;\text{to}\;\\ 2\leq n_{h}\leq N_{h}\;\\ n_{h}\;\text{are}\;\text{integers},\;\forall \;h=1\;\text{and}\;2,\;\;\;\;\vec{n}\varepsilon \nu . \end{array}\right\}$$

We compute payoff matrix of sampler using equation below for various combinations of (*n*_1_, *n*_2_) that satisfy $\vec{n}\varepsilon \nu$

$$\begin{array}{cc} s_{jh}^{2}=\; & \frac{1}{n_{h}-1}\sum_{i=1}^{n_{h}}{\left(y_{jhi}-{\bar{y}}_{jh}\right)}^{2}. \end{array}$$

The above formulation can be expressed as;

$$\left.\begin{array}{c} \;\text{Maximize}\;\;z_{h}=\frac{n_{h}\sum_{i=1}^{n_{h}}{\left(y_{1hi}-{\bar{y}}_{1h}\right)}^{2}}{\left(n_{h}-1\right){\left(\sum_{i=1}^{n_{h}}y_{1hi}\right)}^{2}}+\frac{n_{h}\sum_{i=1}^{n_{h}}{\left(y_{2hi}-{\bar{y}}_{2h}\right)}^{2}}{\left(n_{h}-1\right){\left(\sum_{i=1}^{n_{h}}y_{2hi}\right)}^{2}},h=1\;\text{and}\;2\;\\ \;\text{Subject}\;\text{to}\;\\ 2\leq n_{h}\leq N_{h}\;\\ n_{h}\;\text{are}\;\text{integers},\;\forall \;h=1\;\text{and}\;2,\;\;\;\;\vec{n}\varepsilon \nu . \end{array}\right\}$$

The problem arises where Adversary required a sample of actual values to maximize the sum of $CV_{h}^{2}$ over all characteristics j = 1, 2 for $\vec{n}\varepsilon \nu$. It is feasible under given cost, however, we choose a simulation technique for this purpose. We have sampled more than twice of the total possible samples $N_{h}^{n_{h}}$. The population is known and finite, and sampling is done with replacement for the characteristic vector as well as for all possible sizes under the budgetary restriction. We are able to run a maximum loop on 20 × 10^6^ randomly selected samples. This simulation process returns maximum value of sum of $CV_{h}^{2}$ for both characteristics j = 1, 2 over the whole simulation loop. Results are given in Table 2. Our simulation technique is different from [36], where author simulates thousands of hypothetical populations to identify significant factors while selecting samples under different methodologies.

**Table 2:** Payoff Matrix of sampler.

Adv.’s Strategies →	Stratum 1			Stratum 2			$Min(z_{1}^{*},z_{2}^{*})$
Sampler’s Strategies ↓	$Max\frac{s_{11}^{2}}{n_{1}{\bar{y}}_{11}^{2}}$	$Max\frac{s_{21}^{2}}{n_{1}{\bar{y}}_{21}^{2}}$	Sum	$Max\frac{s_{12}^{2}}{n_{2}{\bar{y}}_{12}^{2}}$	$Max\frac{s_{22}^{2}}{n_{2}{\bar{y}}_{22}^{2}}$	Sum	
2,2	0.065	0.028	0.093	0.028	0.0055	0.0335	0.0349
2,3	0.065	0.028	0.093	0.015	0.0036	0.0186	0.0319
2,4	0.065	0.028	0.093	0.012	0.0036	0.0156	0.0313
2,5	0.065	0.028	0.093	0.012	0.0028	0.0148	0.0311
2,6	0.065	0.028	0.093	0.0105	0.0025	0.013	0.0308
2,7	0.065	0.028	0.093	0.0046	0.0015	0.0061	0.0294
2,8	0.065	0.028	0.093	0.0028	0.0006	0.0034	0.0288
2,9	0.065	0.028	0.093	0.001	0.00005	0.00105	0.0283
3,2	0.040	0.020	0.060	0.028	0.0055	0.0335	0.0249
3,3	0.040	0.020	0.060	0.015	0.0036	0.0186	0.0219
3,4	0.040	0.020	0.060	0.012	0.0036	0.0156	0.0213
3,5	0.040	0.020	0.060	0.012	0.0028	0.0148	0.0211
3,6	0.040	0.020	0.060	0.0105	0.0025	0.013	0.0208
3,7	0.040	0.020	0.060	0.0046	0.0015	0.0061	0.0194
3,8	0.040	0.020	0.060	0.0028	0.0006	0.0034	0.0188
3,9	0.040	0.020	0.060	0.001	0.00005	0.00105	0.0184
4,2	0.030	0.016	0.046	0.028	0.0055	0.0335	0.0207
4,3	0.030	0.016	0.046	0.015	0.0036	0.0186	0.0177
4,4	0.030	0.016	0.046	0.012	0.0036	0.0156	0.0170
4,5	0.030	0.016	0.046	0.012	0.0028	0.0148	0.0169
4,6	0.030	0.016	0.046	0.0105	0.0025	0.013	0.0165
4,7	0.030	0.016	0.046	0.0046	0.0015	0.0061	0.0152
5,2	0.024	0.0125	0.0365	0.028	0.0055	0.0335	0.0178
5,3	0.024	0.0125	0.0365	0.015	0.0036	0.0186	0.0148
5,4	0.024	0.0125	0.0365	0.012	0.0036	0.0156	0.0142
5,5	0.024	0.0125	0.0365	0.012	0.0028	0.0148	0.014
6,2	0.020	0.0105	0.0305	0.028	0.0055	0.0335	0.0160
**6,3**	0.020	0.0105	0.0305	0.015	0.0036	0.0186	**0.0130**

### Solution of minimax game

**Fig 1:**
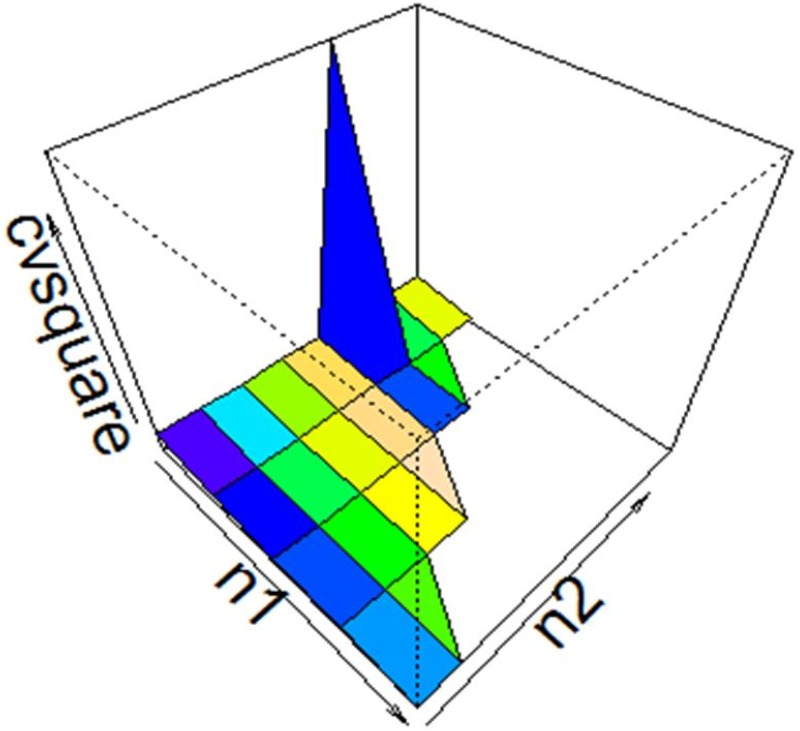
Sampler’s strategies $\vec{n}=\left(n_{1},n_{2}\right)$ vs $Min(z_{1}^{*},z_{2}^{*})$.

For the outer segment of model (7), **we can** use any suitable goal programming technique discussed in ([22, 23, 28], [29–31], [34] and [35]). The above programme Eq (7) can be expressed as a Weighted Goal Programming (WGP) model as;

$$\left.\begin{array}{c} \;\;\text{Minimize}\underset{\vec{n}\;\varepsilon \;\nu }{\;}\;\left(z_{1}^{*},z_{2}^{*}\right)\\ \;\;\text{Subject}\;\text{to}\\ \;c_{0}+\sum_{h=1}^{L}c_{h}n_{h}\leq C\\ \;\sum_{h=1}^{2}n_{h}=n\\ \;n_{h}\;\text{are}\;\text{integers}\;\;\forall \;h=1,2\;\text{and}\;\vec{n}\;\varepsilon \;\nu , \end{array}\right\}$$

where $\left(z_{1}^{*},z_{2}^{*}\right)$ are sum of optimal Adversary’s objectives for stratum 1 and stratum 2, respectively. The objective function in the above equation can be written more precisely as $Minimize\underset{\vec{n}\;\varepsilon \;\nu }{\;}\;\left(z_{1}^{*},z_{2}^{*}\right)=\sum_{h=1}^{L}W_{h}^{2}z_{h}^{*}$, where *W*_*h*_ is *h*^*th*^ stratum weight used in Eq (2). This program runs in general algebraic modeling system (GAMS) to get optimum results. Optimum results are highlighted in Table 2 and visualized in Fig 1.

### Discussion on results

We found for our referred example that the total weighted variation (sum over characteristics) in first stratum ranging from 0.0305 to 0.093, obviously lower for higher sample size. And the same in second stratum is 0.00105 to 0.0335. These results are based on simulation, which may differ on some other attempt [36]. We simulated the results for large number of samples (more than 20 millions in some cases). While comparing fluctuations in two characteristics, it is observed that results show high fluctuation in first characteristic (’test plus interview’ marks) as compare to second (’academic record’ marks) in either strata (see Table 2). The optimal value of this game is 0.01299275 with optimal sampling strategy (6, 3).

In literature, sample selection is frequently discussed when sampling frame is known. But our novel methodology is suitable even if sampling frame is unavailable. This addresses the adverse case scenario while our focus is generally on minimizing the estimates of variation. This sampling strategy shows another side of the picture.

Limitation of this study could be following. First, we have chosen weighted goal programming to solve inner problem of maximization to determine a payoff matrix of sampler. However, one can apply various other methods such as, lexicographic, extended lexicographic, fuzzy programming and the value function technique. Even results may be more interesting for different selection of weight criterion. Second, we use standard weight vector $W_{h}^{2}$ to solve minimax game, however, various other weight vector may be used for outer minimization problem.
